# Investigations on the Potential Role of Free-Ranging Wildlife as a Reservoir of SARS-CoV-2 in Switzerland

**DOI:** 10.3390/v16091407

**Published:** 2024-09-03

**Authors:** Juliette Kuhn, Iris Marti, Marie-Pierre Ryser-Degiorgis, Kerstin Wernike, Sarah Jones, Grace Tyson, Gary Delalay, Patrick Scherrer, Stéphanie Borel, Margaret J. Hosie, Anja Kipar, Evelyn Kuhlmeier, Tatjana Chan, Regina Hofmann-Lehmann, Marina L. Meli

**Affiliations:** 1 Institute for Fish and Wildlife Health, Department of Infectious Diseases and Pathobiology, Vetsuisse Faculty, University of Bern, Länggassstrasse 122, 3012 Bern, Switzerland; iris.marti@unibe.ch (I.M.); patrick.scherrer@wildstation.ch (P.S.);; 2 Institute of Diagnostic Virology, Friedrich-Loeffler-Institut (FLI), Südufer 10, 17493 Greifswald-Insel Riems, Germany; kerstin.wernike@fli.de; 3 School of Biodiversity, One Health, and Veterinary Medicine, College of Medical, Veterinary and Life Sciences, University of Glasgow, Bearsden Road, Glasgow G61 1QH, UK; 4 MRC-University of Glasgow Centre for Virus, College of Medical, Veterinary and Life Sciences, University of Glasgow, Garscube Campus, Bearsden Road, Glasgow G61 1QH, UK; 5 Institute of Veterinary Pathology, Vetsuisse Faculty, University of Zurich, Winterthurerstrasse 268, 8057 Zurich, Switzerland; anja.kipar@uzh.ch; 6 Clinical Laboratory, Department of Clinical Diagnostics and Services, and Center for Clinical Studies, Vetsuisse Faculty, University of Zurich, Winterthurerstrasse 260, 8057 Zurich, Switzerland; evelyn.kuhlmeier@web.de (E.K.); tatjana.chan@uzh.ch (T.C.); regina.hofmann-lehmann@uzh.ch (R.H.-L.); mmeli@vetclinics.uzh.ch (M.L.M.)

**Keywords:** one health, spillover, zoonosis, fox, lynx, wildcat, S1-ELISA, RBD-ELISA, indirect immunofluorescence test, surrogate virus neutralization test, pseudotype-based virus neutralization assay

## Abstract

Amid the SARS-CoV-2 pandemic, concerns surfaced regarding the spread of the virus to wildlife. Switzerland lacked data concerning the exposure of free-ranging animals to SARS-CoV-2 during this period. This study aimed to investigate the potential exposure of Swiss free-ranging wildlife to SARS-CoV-2. From 2020 to 2023, opportunistically collected samples from 712 shot or found dead wild mustelids (64 European stone and pine martens, 13 European badgers, 10 European polecats), canids (449 red foxes, 41 gray wolves, one golden jackal) and felids (56 Eurasian lynx, 18 European wildcats), as well as from 45 captured animals (39 Eurasian lynx, 6 European wildcats) were tested. A multi-step serological approach detecting antibodies to the spike protein receptor binding domain (RBD) and N-terminal S1 subunit followed by surrogate virus neutralization (sVNT) and pseudotype-based virus neutralization assays against different SARS-CoV-2 variants was performed. Additionally, viral RNA loads were quantified in lung tissues and in oronasal, oropharyngeal, and rectal swabs by reverse transcription polymerase chain reactions (RT-qPCRs). Serologically, SARS-CoV-2 exposure was confirmed in 14 free-ranging Swiss red foxes (prevalence 3.1%, 95% CI: 1.9–5.2%), two Eurasian lynx (2.2%, 95% CI: 0.6–7.7%), and one European wildcat (4.2%, 95% CI: 0.2–20.2%). Two positive foxes exhibited neutralization activity against the BA.2 and BA.1 Omicron variants. No active infection (viral RNA) was detected in any animal tested. This is the first report of SARS-CoV-2 antibodies in free-ranging red foxes, Eurasian lynx, and European wildcats worldwide. It confirms the spread of SARS-CoV-2 to free-ranging wildlife in Switzerland but does not provide evidence of reservoir formation. Our results underscore the susceptibility of wildlife populations to SARS-CoV-2 and the importance of understanding diseases in a One Health Concept.

## 1. Introduction

COVID-19, caused by SARS-CoV-2 infection, is a viral disease that is most likely of animal origin (bat [1,2]) and has become a pandemic following unclear viral spillover events. Worldwide, as of February 11, 2024, in humans, more than 774,631,444 confirmed cases of COVID-19, including 7,031,216 deaths, have been reported to the WHO [3]. Although the primary source of infection in the pandemic has been human-to-human transmission, cases of SARS-CoV-2 infection in animals are still occurring [4,5]. As of March 2024, 909 outbreaks in animals have been reported globally, affecting 35 species in 40 countries [4,6]. SARS-CoV-2 has been detected in companion animals (dogs, cats, ferrets, and hamsters), captive wildlife (tigers, lions, snow leopards, cougars, lynx, fishing cats, binturongs, hyenas, otters, coatimundi, hippopotamuses, white-tailed deer, and gorillas), farmed animals (minks, cattle, and horses), and free-ranging wildlife (white-tailed deer, mule deer, otters, and minks) [4,7,8,9]. This raises concerns, since the establishment of SARS-CoV-2 reservoirs in animals and possible spillbacks to humans could pose a serious health risk.

In red foxes (*Vulpes vulpes*), experimental exposure led to infection and virus shedding [10]. Juvenile red foxes proved to be susceptible to SARS-CoV-2 following experimental instillation of the cell-cultured virus into their nares. The virus was shed orally and nasally by all six foxes in the study for up to three days before ceasing by day five. Upon necropsy, no gross lesions were observed in the animals. A study conducted in Croatia in 2020/2021 found that 2.9% of free-ranging wild red foxes and 4.6% of jackals (*Canis aureus moreoticus*) tested positive for antibodies against SARS-CoV-2 in a commercial ELISA test using nucleocapsid (N) protein, but these results were not validated by sVNT. The authors concluded that there was no spillover event [11]. In 2020, in China 89 foxes tested negative using an ELISA based on the S1 protein [12]. Recently, SARS-CoV-2 RNA was detected from an oropharyngeal swab in a park-kept captive fox in Switzerland [13,14,15]. In captivity, a Eurasian lynx (*Lynx lynx*) from the Zagreb Zoo Park [16] and a Canadian lynx (*Lynx canadensis*) from a zoo in Pennsylvania [17] tested positive for SARS-CoV-2 and showed clinical respiratory signs. To our knowledge, natural infections in free-ranging red foxes, European wildcats, and Eurasian lynx have so far not been reported.

Animals, like humans, are susceptible to SARS-CoV-2 and can manifest severe illness, raising concern that wildlife could act as a reservoir for the virus. However, from an animal welfare and wildlife conservation perspective, the transmission of the virus from humans to animals, eventually leading to their morbidity, is equally worrying. A broad spectrum of clinical manifestations following infection has been documented in various species, including minks (*Neovison vison*) [18], mice (*Mus musculus*), ferrets (*Mustela putorius furo*) [19], cats (*Felis catus*) [19,20,21], and lions (*Panthera leo*) [22].

In Ohio, USA, the susceptibility of free-ranging white-tailed deer (*Odocoileus virginianus*) to SARS-CoV-2 infection was observed. During the period from January to March 2021, over one-third of nasal swabs obtained from the deer tested positive for SARS-CoV-2. The deer exhibited infections with multiple SARS-CoV-2 variants (B.1.2, B.1.582, and B.1.596), with evidence of potential deer-to-deer transmission [9].

These cases underscore the complex dynamics of SARS-CoV-2 infections across different animal species. Due to the close contact between wildlife, domestic animals, and humans, SARS-CoV-2 must be considered in a “One Health” approach. Potentially susceptible species must be continuously monitored and documented.

The objective of this study was to determine the potential exposure of free-ranging Swiss wildlife to SARS-CoV-2. The study focused on two highly susceptible animal families, mustelids and felids. Moreover, canids were included, since, e.g., foxes often come into close contact with humans and inhabit crowded urban areas and therefore can be assumed to have an increased exposure risk. The study determined sample seroprevalence using SARS-CoV-2 antibody binding and surrogate neutralizing assays from blood samples. Moreover, swabs (oronasal/oropharyngeal and rectal swabs) and tissue samples (lung tissue) were tested for viral RNA using two SARS-CoV-2 RT-qPCR assays, and, in one case, for viral antigen expression in the lungs, by immunohistology.

## 2. Materials and Methods

### 2.1. Study Set-Up

For the present project, we tested free-ranging wildlife native to Switzerland. These included wild felids, namely the Eurasian lynx (*Lynx lynx*) and the European wildcat (*Felis silvestris*), mustelids of the subfamilies Mustelinae (*Martes foina*, *M. martes*, *Mustela putorius*, *M. ermine*, *M. nivalis*) and Melinae (*Meles meles*), and canids (*Vulpes vulpes*, *Canis lupus*, *Canis aureus*).

Sampling approaches comprised opportunistic sampling during postmortem examinations, sampling of dead animals, and sampling of live-captured animals in the field. The sampled species included animals living in social groups in urbanized areas with close contact to human settlements, such as foxes, as well as elusive solitary species such as lynx. Species, social behavior, sampling method, and the number of samples are summarized in Table 1.

The target sample size for non-protected, hunted species was calculated for red foxes and mustelids (badgers and martens), assuming simple random sampling. The free online tool by AusVet Animal Health Services (https://epitools.ausvet.com.au, accessed on 1 September 2021) was used for the calculation, applying the method for the estimation of true prevalence assuming perfect test characteristics. Design prevalence was set at 50% because no prior information on the prevalence of infection in indigenous species was available. The population size was considered infinite for foxes (i.e., >20,000), >12,000 for badgers, and >5000 for stone and pine martens based on yearly hunting bags of 20,000 for foxes, 4000 for badgers, and 1500 for martens (www.jagdstatistik.ch, accessed on 1 September 2021). Precision was set at 5%, and the level of confidence was set at 95%. The calculated sample size using serological assessment was 500 for the red fox and 400 for the mustelids. For the sampling campaign, the obtained sample size per taxon was distributed among the 26 Swiss cantons to ensure an even sample distribution across the country, i.e., including cantons with and without an international border and with different levels of urbanization.

### 2.2. Sample Collection

#### 2.2.1. Postmortem Examinations

The Institute for Fish and Wildlife Health (FIWI) conducts the Swiss national general wildlife health surveillance. Between 2021 and 2023, 176 animals submitted for diagnostic postmortem examination were sampled for this study (Table 1). While all lynx, wildcats, wolves, stone martens, pine martens, badgers, polecats, and golden jackals were sampled, sampling of red foxes was restricted to animals with an anamnesis of contact with human settlements (found dead or culled ≤2000 m from a human settlement). Collected material included oronasal swabs (n = 165), rectal swabs (n = 163), lung tissue (n = 160), and blood (n = 168). The sampling took place in the necropsy room at FIWI and was performed by the veterinary pathologists on duty. For this sampling, we used cotton swabs with a plastic stem (Divers Dutscher; Bernolsheim, France; 020310). The swabs were then introduced into 1.5 mL Eppendorf tubes (Eppendorf AG, Hamburg, Germany) filled with 400 µL of DNA/RNA shield solution (Zymo Research Europe GmbH, Freiburg, Germany). The lung tissue was put into 2 mL screw cap tubes (Sarstedt AG & Co., Nümbrecht, Germany) filled with 600 µL DNA/RNA shield solution. All samples were stored at −20 °C until further analysis. Gloves and a surgical mask were systematically worn throughout the sampling process to avoid contamination of the samples.

#### 2.2.2. Field Sampling of Culled Animals or Found Dead Animals

Sampling sets were prepared and sent to 24 hunting authorities in 23 Swiss cantons and the Principality of Liechtenstein. These sets included sampling instructions, blood collection tubes (Sarstedt AG & Co., serum tube, 10 mL, REF 26.367), nitrile gloves (Meditrade^®^, Kiefersfeld, Germany), 10 mL syringes, and a questionnaire. The local field partners (game wardens, hunters, veterinarians) collected blood from 473 animals, including red foxes; European pine and stone martens; European badgers; and European polecats that were either culled, found dead, or died in a rehabilitation center.

Blood samples were sent with priority shipping (1–2 days) to the FIWI and centrifuged on the day of arrival; serum was stored at −20 °C until analysis. Additionally, a questionnaire was filled out with data on the proximity to human households, waste, or domestic animals (cats, dogs, livestock) and on the sampled animals (species, sex, age). The general health status was determined by field inspection performed by the game warden or hunters.

#### 2.2.3. Field Sampling of Captured Felids

In addition to the planned sample collections during necropsies and by field sampling, samples became available during wildlife immobilization. Veterinarians of the FIWI participated in lynx capture campaigns from 2020 to 2023 and wildcat capture campaigns from 2021 to 2023 as part of an international translocation project for lynx and a project investigating the recolonization of Switzerland by the European wildcat. All captured animals were clinically examined and sampled (heparin anticoagulated and native whole blood; oropharyngeal and rectal dry swabs) for hematology and molecular testing to assess their health status. Native whole blood samples were centrifuged on the day of capture. Serum and swabs were then stored at −20 °C until further analysis. Overall, 46 blood samples, 44 oropharyngeal swabs, and 44 rectal swabs from 39 lynx and 6 wildcats were available for this study. Two blood samples were taken from one monitored lynx (F23_15), on two different days, 23 February 2023, and 2 March 2023, and both samples were tested.

#### 2.2.4. Overview of Animals and Samples Included in the Study

In total, 757 animals were sampled between 27 October 2020, and 24 March 2023 (Table 1).

For the serological analyses, blood from 746 animals was available. In total, for the molecular analyses, oronasal (n = 165), oropharyngeal (n = 45), and rectal swabs (n = 207), as well as lung tissue (n = 162), from 176 necropsied animals and 45 captured animals were available (Table 2).

**Figure 1:**
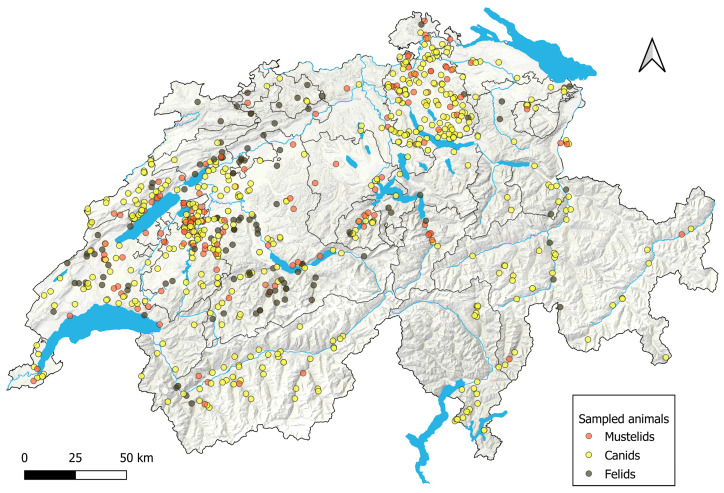
Distribution of all sampled animals in the different cantons of Switzerland and in Liechtenstein. Grey shades show land features, blue indicates rivers and lakes, and grey lines mark canton boundaries on the QGIS-designed map. Each dot on the map represents one sampled animal. Visualization of the data was performed using the Quantum Geographic Information System (QGIS 3.28.3—Firenze version, Open Source Geospatial Foundation Project (http://qgis.org, accessed on 1 May 2024).

Samples were collected from all 26 Swiss cantons and the Principality of Liechtenstein. In Figure 1, the distribution of samples is depicted on a geographical map. The exact number of sampled animals per canton in Switzerland and in the Principality of Liechtenstein can be found in Table S3.

### 2.3. Serological Analyses

The native whole blood samples were centrifuged for 20 min at 3500 rpm at 4 °C upon arrival at the FIWI, and serum was collected in separate tubes and stored at −20 °C until further analysis. Serum samples were analyzed for the presence of antibodies. The level of hemolysis in the serum samples was graded on a scale of 0 to 3 based on the intensity of red coloration, with 0 indicating non-hemolysed and 3 indicating highly hemolysed samples. All sera were heat-inactivated at 56 °C for 1 h prior to analysis.

**Figure 2:**
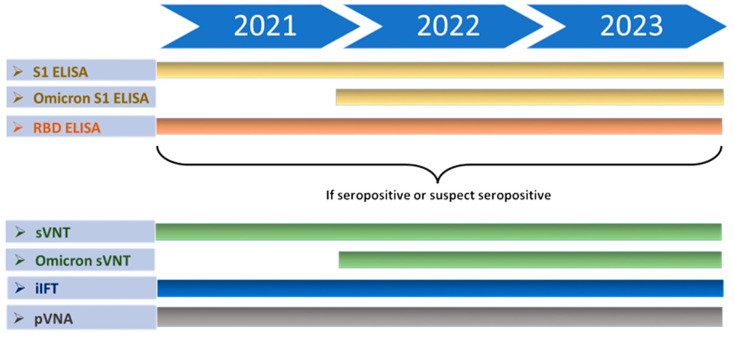
Timeline of serological analyses. Samples with ODs above the cutoff values (≥mean + 3SD) and those exhibiting high ODs under the cutoff values (≥mean + 2SD) in ELISA underwent further testing with sVNT, iIFT, and PVNA.

Samples were tested following the concept displayed in Figure 2; details about the tests are provided below. All serum samples were first screened with the SARS-CoV-2 S1-ELISA. Samples collected between 2022 and 2023 were additionally tested with the Omicron S1-ELISA. Moreover, all serum samples from felids and canids were tested using an RBD-ELISA. Results were validated by indirect immunofluorescence tests (iIFT) at the Friedrich-Loeffler-Institut (FLI). A Surrogate Virus Neutralization Test (sVNT) and a Pseudotype-Based Virus Neutralization Assay (PVNA) were performed at the University of Zurich (UZH) and the University of Glasgow, respectively, to assess neutralizing activity against SARS-CoV-2 in positive or suspect positive samples.

For all serological tests (ELISA, sVNT, iIFT, PVNA), a panel of pre-COVID samples from the biobank of the Clinical Laboratory (Department of Clinical Diagnostics and Services, Vetsuisse Faculty, University of Zurich) was tested for each concerned species. The pre-COVID samples from each species were used to calculate the mean concentration (MC) and cut-off values. Serologically, animals were considered suspect positive for SARS-CoV-2 binding antibodies if they were seropositive for binding antibodies (cutoff values ≥ mean + 3SD) in at least one of the ELISAs (RBD, S1 or Omicron S1). They were confirmed positive for binding antibodies if validated by iIFT (Friedrich-Loeffler-Institut). Moreover, samples were considered seropositive if they contained neutralizing antibodies in sVNT or PVNA (University of Glasgow).

#### 2.3.1. SARS-CoV-2 S1 and Omicron S1 Enzyme-Linked Immunosorbent Assay (ELISA)

The SARS-CoV-2 S1-ELISA was adapted from a previously described protocol [23]. Briefly, the proteins SARS2-S1-3ST (for S1-ELISA, kindly provided by Prof. Dr. Herman Egberink, Utrecht University) or S1 Protein Omicron (SARS-CoV-2 Spike protein S1, Omicron Variant, (His Tag, Z03729-100, GenScript Inc., EG Rijswijk, the Netherlands) for Omicron S1-ELISA) were prepared in Dulbecco’s Phosphate Buffered Saline (DPBS, pH 7.4, with Ca^2+^/Mg^2+^). We used 96-well microtiter plates (Greiner-Bio One, St. Gallen, Switzerland) which were coated with 100 µL of the antigen solution to achieve a concentration of 1 pmol per well (S1-ELISA) or 20 ng per well (Omicron S1-ELISA), and they were incubated overnight at 4 °C. Plates were subsequently washed with a Wellwash Microplate Washer (Thermo Fisher Scientific, Basel, Switzerland) using Phosphate Buffered Saline (PBS, Life Technologies Ltd., Paisley, UK) with 0.05% Tween-20 (H5152, Promega AG, Dübendorf, Switzerland).

To reduce non-specific binding, 200 µL blocking buffer containing PBS, 5% milk powder (B501-0500, Bioconcept, Allschwil, Switzerland), and 0.05% Tween-20 (Promega AG) was added to each well and incubated for 2 h at 37 °C.

Serum samples and controls were diluted in the blocking buffer at a 1:50 ratio by mixing 588 µL of blocking buffer with 12 µL of serum sample. As a positive control for the felid samples, serum of a domestic cat that had previously tested positive was used (animal 25607, [20]); for the canid samples, two positive dog sera (USZ6 animals 4 and 5, [24]) were used and mixed to a 50:50 ratio; for the mustelid, serum of a positive ferret (provided by Prof. Dr. Martin Beer, Friedrich-Loeffler-Institut) was used. Pre-COVID sera from the respective species served as negative controls. After washing the plates, 100 µL of the serum dilutions as well as appropriate species-specific positive and negative controls were added to each well in duplicate and incubated for 1 h at 37 °C. The plates were then washed again as described above.

For conjugation, 100 µL of the species-specific secondary antibodies at a 1:6000 dilution in blocking buffer were added and incubated for 1 h at 37 °C. For canid samples (foxes, wolves, golden jackal), a rabbit anti-dog immunoglobulin (IgG) horseradish-peroxidase (HRP)-conjugated secondary antibody (Jackson ImmunoResearch Europe, Ely, UK) was used; for felid samples (wildcats, lynx), a goat anti-cat IgG (H + L) secondary antibody (Jackson ImmunoResearch Europe) was used; and for human samples (positive control for Omicron), a goat anti-human IgG HRP (Abcam, Cambridge, UK) was used.

For mustelid samples (polecats, badgers, pine martens, stone martens), an anti-multi-species IgG-HRP conjugate (ID Screen^®^ Schmallenberg virus Milk Indirect, IDVet, Grabels, France) was used and diluted at 1:40 in blocking buffer as described previously [25].

After an additional wash, 100 µL of 3,3′,5,5′-tetramethylbenzidine substrate (BioFX TMB Super Slow, TTMB-1000-01, LubioScience GmbH, Zurich, Switzerland) was added to each well and incubated at room temperature for 5 min, shielded from direct light. The reaction was stopped using 100 µL of 2 M H_2_SO_4_ (BioFX Liquid Nova Stop for TMB Substrates, NSTP-1000-01, LubioScience GmbH). Optical density (OD) was measured using a spectrophotometer (SPECTRAmax PLUS 384, Molecular Devices LLC, San Jose, CA, USA) at 450 nm. The standardized OD was calculated as (OD value [sample]—OD value [negative control]/(OD value [positive control]—OD value [negative control]). The cut-off values for suspect positive samples were set at three-fold standard deviations above the mean value of the reactivity of samples from a pre-COVID-19 cohort (samples collected before 2020) for each animal species tested.

#### 2.3.2. SARS-CoV-2 RBD-ELISA

Serum samples were tested by using an in-house established ELISA to detect antibodies binding to the SARS-CoV-2 spike glycoprotein receptor-binding domain (RBD), as previously described [26,27,28], with species-specific modifications. Briefly, the recombinant SARS-CoV-2 Spike Protein RBD, Wuhan-Hu-1 (LU2020, LubioScience GmbH), was prepared to achieve a concentration of 200 ng/well. ELISA plates (Greiner-Bio One, St. Gallen, Switzerland) were coated with 100 µL of the antigen and incubated for 3 h at 37 °C and overnight at 4 °C. Between all steps, the plates were washed three times with ELISA wash buffer (pH 7.4, 0.15 M sodium chloride, 0.2% Tween 20 (Sigma-Aldrich Chemie GmbH, Buchs, Switzerland)) and tapped dry. The same positive and negative controls were used as described above for S1-ELISA.

All sera were diluted at 1:100 in buffer P3X (8.7 g (0.15 M) sodium chloride, 372 mg (1 mM) Titriplex III, 6.05 g (0.05 M) Tris(hydroxymethyl)aminomethane, 1 g BSA, 1 g Tween in 900 mL ddH_2_O, pH 7.4 adjustment with 1 M HCl, total volume to 1 L with ddH_2_O), pipetted to each plate in a total volume of 100 µL/well, and incubated at 37 °C for 1 h. Each sample and all controls were run in duplicate. The conjugate dilutions of the previously described secondary antibodies were prepared as follows: 1:3000 (goat anti-cat IgG, rabbit anti-dog IgG) or 1:40 (anti-multi-species IgG [25]) in P3X, and 100 µL/well was used. A substrate solution containing ABTS (Sigma-Aldrich) was pipetted into each well. The plates were read after 10 min using a spectrophotometer (SPECTRAmax PLUS 384, Molecular Devices LLC) at an optical density of 415 nm. The standardized OD was calculated as in Section 2.3.1. The cut-off values for suspect positive samples were set at three-fold standard deviations above the mean value of the reactivity of samples from a pre-COVID-19 cohort (samples collected before 2020) for each animal species tested.

#### 2.3.3. Indirect Immunofluorescence Test (iIFT)

For confirmatory testing, samples suspected of being positive or suspect positive by either RBD-ELISA, S1-ELISA, or Omicron S1-ELISA were first sent to the Friedrich-Loeffler-Institut. Confirmation was performed there by indirect immunofluorescence test (iIFT) according to an established protocol [2,29].

In short, viral infection was performed in a Biosafety Level 3 (BSL3) containment area, with fixation at 24 h post-infection using 4% paraformaldehyde (PFA) in PBS, at a volume of 100 µL per well for 15 min at room temperature.

The fixed cells were permeabilized using 0.5% Triton X-100 in PBS for 10 min, followed by two washes with TBST (Tris-buffered saline, 0.1% Tween 20). Serum application involved adding diluted sera to the wells (virus-infected cells and uninfected cells for control, 50 µL per well), starting with a dilution of 1/8 or 1/16 based on the serum sample availability, and incubating for 1 h. For secondary antibody staining, cells were washed twice with TBST post-serum application and then stained with the corresponding FITC-conjugated secondary antibodies, including FITC anti-goat (1:200, 11839170, Invitrogen), FITC anti-dog (1:100, F4012-2ML, Sigma-Aldrich), FITC anti-cat (1:600, SAB3700056-2MG, Sigma-Aldrich), or FITC anti-ferret (1:50, SAB3700800, Sigma-Aldrich).

Following incubation with the secondary antibodies, cells underwent a final wash with TBST and were overlaid with 50 µL of DABCO per well to preserve fluorescence.

Samples with titers greater than 1:8 were considered positive.

#### 2.3.4. Surrogate Virus Neutralization Test (sVNT)

In case of suspect positive or positive results from the RBD-ELISA, S1-ELISA, or Omicron S1-ELISA, samples were tested by a commercially available SARS-CoV-2 Surrogate Virus Neutralization Test Kit (sVNT; GenScript). All samples from 2022 and 2023 were additionally tested with an Omicron variant of the sVNT Kit. Briefly, the following reagents were used instead of those provided by the kit: SARS-CoV-2 Spike protein RBD-HRP, Omicron Variant, His Tag (Z03730-100, GenScript), and as positive control antibody, the SARS-CoV-2 (Omicron) Neutralizing Antibody Standard (A02161-100, GenScript). These tests detect neutralizing activity against SARS-CoV-2 RBD of the spike protein by antibodies in the sera that prevent the binding of RBD to the ACE2 receptor. The tests were performed according to the manufacturer’s protocols. Positive and negative controls were provided in the kits.

Optical density was measured in a spectrophotometer (SPECTRAmax PLUS 384, Molecular Devices LLC) at 450 nm. The percentage of inhibition was then calculated with the following formula [30]:

Inhibition (%) = (1 − OD value of sample/OD value of negative control) × 100

The cut-off values for suspect positive samples were set at three-fold standard deviations above the mean value of the reactivity of samples from a pre-COVID-19 cohort (samples collected before 2020) for each animal species tested.

#### 2.3.5. Pseudotype-Based Virus Neutralization Assay (PVNA)

Samples with suspect positive results in ELISA were also sent to the University of Glasgow (Centre for Virus Research) for pseudotype-based virus neutralization assays (PVNA), for confirmatory testing. The assay method, previously described [31,32,33,34], involved culturing HEK293T and HEK293-ACE2 cells in Dulbecco’s modified Eagle’s medium (DMEM), supplemented with 10% fetal bovine serum, 200 mM L-glutamine, 100 µg/mL streptomycin, and 100 IU/mL penicillin (“complete DMEM”). In short, HEK293T cells were transfected with the corresponding SARS-CoV-2 S gene expression vector (wild type or variant: Wuhan/B.1, Alpha, Delta, Omicron, BA.2, BA.5, BQ.1.1, XBB) to produce HIV (SARS-CoV-2) pseudotypes which were harvested, filtered, aliquoted, and frozen at −80 °C prior to use. HEK293-ACE2 target cells were maintained in complete DMEM supplemented with 2 µg/mL puromycin. Samples were incubated for 1 h with each pseudotype at a single sample dilution of 1:50 in complete DMEM and then plated onto HEK293-ACE2 target cells. Following an incubation of 48–72 h, luciferase activity was measured. When the samples contained neutralizing antibodies, luciferase activity was reduced as the pseudotypes were prevented from penetrating the cells. Sera with ≥90% reduction in infectivity, compared to a no serum control, were considered to be positive. For samples with a positive result for at least one pseudotype, neutralizing antibody titers were obtained by repeating the assay with serially diluted samples to estimate antibody titer based on infectivity reduction. The titration assay was performed in triplicate. The titer was then defined as the dilution factor which reduced the infectivity by 90%, in comparison to a no serum control. A control test was performed using a ninth pseudotype, featuring the same lentiviral backbone as the SARS-CoV-2 pseudotypes but bearing vesicular stomatitis virus glycoprotein-G (VSV-G) rather than SARS spike proteins.

### 2.4. Immunohistology

Formalin-fixed and paraffin-embedded lung samples from a lynx (W23_0441) were analyzed at the Institute of Veterinary Pathology (Vetsuisse Faculty Zürich) for the in situ detection of SARS-CoV-2 nucleoprotein by immunohistochemistry, following a published protocol [35].

### 2.5. Molecular Detection of SARS-CoV-2

Viral RNA was extracted from oronasal and rectal swabs and lung tissues and analyzed using SARS-CoV-2 RT-qPCRs.

#### 2.5.1. Nucleic Acid Extraction

Swab samples were incubated at 42 °C for 30 min in a shaking incubator at 600 rpm. Following this, the tubes with cotton swabs were centrifuged at 8000 rpm for 1 min, as described in [20]. Cotton swabs were inverted within the tubes using tweezers, which were cleaned between each sample with RNAse Away (Thermo Fisher Scientific) and then with 70% ethanol. These tubes underwent another centrifugation at 8000 rpm for 1 min. Using the cleaned tweezers as described above, the cotton swabs were then removed. Total Nucleic Acid (TNA) extraction was performed using the MagNA Pure LC 2.0 and the MagNa Pure high-performance Total Nucleic Acid Kit (Roche Diagnostics AG, Rotkreuz, Switzerland) following the manufacturer’s instructions. With each extraction batch, a negative control consisting of PBS (Life Technologies Ltd.) was extracted in parallel.

For the lung tissue samples, RNA was isolated using the QIAGEN RNeasy mini kit (Qiagen, Hilden, Germany). Approximately 50 mg of lung tissue was placed into 700 µL of RLT buffer (Qiagen, Hilden, Germany) that included beta-hydroxybutyrate in a 2 mL Precellys^®^ CK14 tube (Bertin Technologies SAS, Montigny-le-Bretonneux, France). Tissue samples were then homogenized using a Precellys^®^ 24 tissue homogenizer (Bertin Technologies SAS) for 2 × 30 s at a speed of 5000/min. A total of 650 µL of the liquid was transferred to a QIAshredder spin column (Qiagen, Hilden, Germany) and was centrifuged for 2 min, after which RNA was extracted according to the manufacturer’s instructions. In each extraction batch, a negative control of PBS (Life Technologies Ltd.) was used. All TNA, RNA, and the negative extraction controls were then stored at −20 °C before further testing.

#### 2.5.2. SARS-CoV-2 RT-qPCRs

For the detection of SARS-CoV-2 RNA, TNA from oronasal and rectal swabs as well as RNA from lung tissues were first screened by RT-qPCR targeting the viral envelope gene (E). Samples with questionable or positive results were subsequently tested using RT-qPCR targeting the RdRp gene as previously described [20,36]. The TNA was tested both neat and diluted (1:5 with RNAse-DNase free water (AppliChem, Darmstadt, Germany)) to monitor for possible inhibition. In each RT-qPCR, a negative control (RNase-DNase free water) and the negative extraction control, as well as a positive control (in vitro-transcribed RNA control containing three concatenated sequences of RdRp, E, and nucleocapsid (N) SARS-CoV-2 genes: RNA_Wuhan_RdRp-E-N; kindly provided by the Swiss Federal Institute for Virology and Immunology, Mittelhäusern, Switzerland) were run in parallel. All assays were performed on an ABI PRISM 7500 Fast Instrument (Applied Biosystems, Foster City, CA, USA) using 4 µL of TNA and the TaqMan^®^ Fast Virus 1-Step Master Mix (Applied Biosystems) in an RT-qPCR protocol as previously described [24]. The RT-qPCR test was considered negative when the cycle threshold (Ct) value was ≥45.

### 2.6. Submission to ProMed and OIE

Seropositive animals were officially reported to the World Organization for Animal Health (WOAH) [15] and to ProMED [13], as well as to the Swiss Federal Office for the Environment (BAFU) and the Federal Food Safety and Veterinary Office (BLV).

### 2.7. Statistical Analysis and Software

Confidence intervals for sample prevalence (CI) were calculated using R Statistical Software (v4.1.2; R Core Team 2021) and the Wilson score interval with a confidence interval of 95% (Hmisc package, binconf function). Serology data were displayed by the box plot method with R Statistical Software (v4.1.2; R Core Team 2021) and the ggplot2 package.

The distribution of samples on geographical maps was visually represented using the Quantum Geographic Information System (QGIS 3.28.3—Firenze version, Open Source Geospatial Foundation Project. http://qgis.org, accessed on 1 May 2024).

Additionally, the Grammar Checker DeepLWrite (https://www.deepl.com/de/write, accessed on 1 June 2024) was employed to refine the grammatical aspects of the content.

## 3. Results

### 3.1. Serological Analyses

Serologically, animals were considered suspect positive for SARS-CoV-2 binding antibodies if they were seropositive for binding antibodies (cutoff values ≥ mean + 3SD) in at least one of the ELISAs. Of the 746 animals analyzed (Table 2), 77 animals tested suspect positive (10.5%, 95% CI: 8.5–12.9%). This included 64/446 red foxes (14.3%, 95% CI: 11.4–17.9%), 3/92 Eurasian lynx (3.3%, (95% CI: 1.1–9.2%), 1/10 European polecats (10%, 95% CI: 0.5–40.4%), 1/48 stone martens (2.1%, 95% CI: 1.1–10.9%), 6/23 European wildcats (26.1%, 95% CI: 12.5–46.5%), and 2/41 grey wolves (4.9%, 95% CI 1.3–16.1%). None of the 72 European badgers tested suspect positive, nor did the 13 tested pine martens and the golden jackal (Table 3).

Fifteen of the 78 suspect positive samples were confirmed positive for binding antibodies through IIFT, including 12 red foxes and 3 Eurasian lynx. Additionally, four samples tested positive for neutralizing antibodies (PVNA or sVNT), comprising two red foxes, one European wildcat, and one Eurasian lynx. The comprehensive list of animals identified as suspect positive or confirmed positive, along with their corresponding test results, is available in Table S1 for reference.

#### 3.1.1. Foxes

Among the 446 fox blood samples, 64 tested suspect positive for binding antibodies (14.3%, 95% CI: 11.4–17.9%). Thereof, 7 tested positive in the RBD-ELISA, 13 tested positive in the S1-ELISA, and 51 tested positive in the Omicron-S1-ELISA. A total of 12/64 samples were confirmed for binding antibodies through iIFT (Table 3 and Table 4, Figure S1).

**Figure 3:**
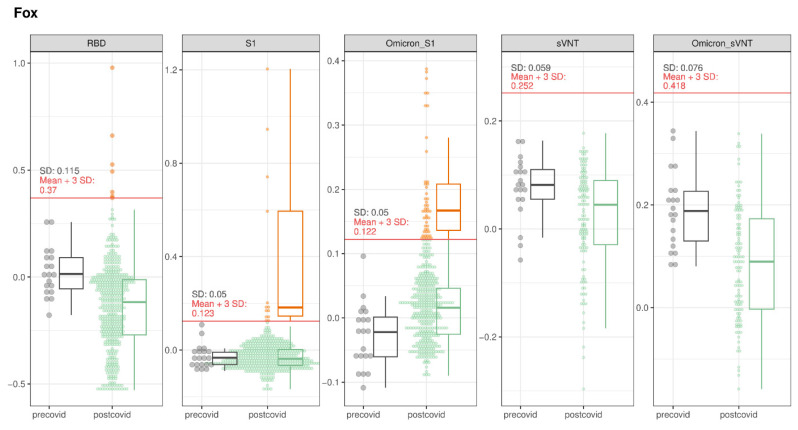
SARS-CoV-2 serological results for foxes tested with RBD-, S1-, and Omicron S1-ELISA, as well as with sVNT and Omicron sVNT. The OD values of the different assays for the pre-COVID and post-COVID populations are represented. The boxes cover the 25th to 75th percentiles, while the whiskers represent the upper and lower 25th percentiles. For populations with less than 10 animals, values were plotted as points with a small jitter effect on the *y*-axis to prevent overplotting. When the population exceeded 10 animals, values were depicted using both dot plots and box plots. A cutoff, defined as the sum of the mean values for the pre-COVID population and three times the standard deviation, is denoted by a horizontal red line on the figure.

**Figure 4:**
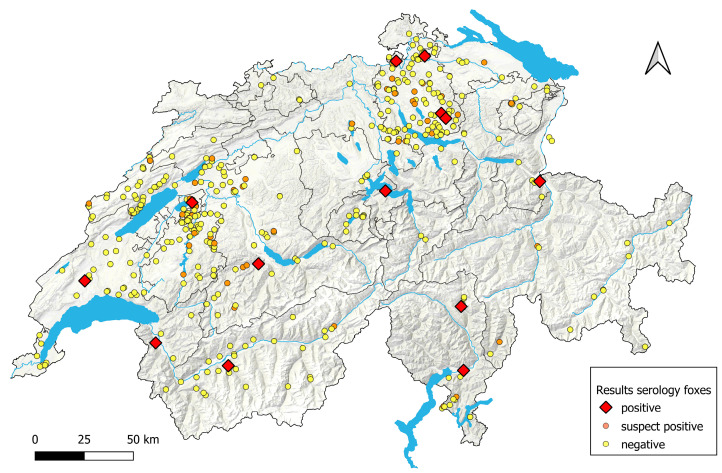
Distribution of serologically positive, suspect positive, and negative foxes in the different cantons of Switzerland and Liechtenstein. Grey shades show land features, blue indicates rivers and lakes, and grey lines mark canton boundaries on the QGIS-designed map. Each dot on the map represents one sampled animal.

None of the samples tested positive in the sVNT or Omicron sVNT (Figure 3). Additionally, two samples (#1577 and #371) were determined to have neutralizing antibodies through PVNA, with higher inhibition titers for the variants BA.2 (sample #1577) and Omicron BA.1 (sample #371) (Table S2), showing a combined prevalence for confirmed infected animals for both binding and neutralizing antibodies of 3.1% (95% CI: 1.9–5.2%). Inhibition titers in PVNA across the different variants were similar (Table S2). All 14 animals had been found within 500 m of human settlements (Figure 4).

The distribution of the 14 confirmed seropositive and the 50 suspect positive foxes is shown on the map in Figure 4.

#### 3.1.2. Lynx

**Figure 5:**
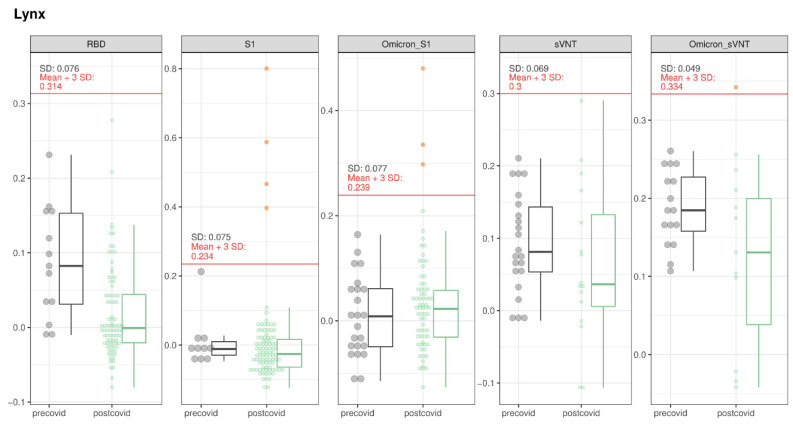
SARS-CoV-2 serological results for lynx tested with RBD-, S1-, and Omicron S1-ELISA, as well as with sVNT and Omicron sVNT. The OD values of the different assays for the pre-COVID and post-COVID populations are represented. The boxes cover the 25th to 75th percentiles, while the whiskers represent the upper and lower 25th percentiles. For populations with less than 10 animals, values were plotted as points with a small jitter effect on the *y*-axis to prevent overplotting. When the population exceeded 10 animals, values were depicted using both dot plots and box plots. A cutoff, defined as the sum of the mean values for the pre-COVID population and three times the standard deviation, is denoted by a horizontal red line on the figure.

All 91 lynx tested negative in RBD-ELISA. Samples originating from three animals (F23_15, F22_21, W23_0441) tested suspect positive for binding antibodies in the S1-ELISA. Samples from F23_15 and W23_0441, collected in 2023, also tested suspect positive in the Omicron S1-ELISA (Figure 5). This resulted in a prevalence of suspect positive animals of 3.3%, (95% CI: 1.1–9.2%). Samples from F23_15 and W23_0441 were confirmed for binding antibodies through iIFT, indicating a prevalence for positive animals of 2.2% (95% CI: 0.6–7.7%).

**Figure 6:**
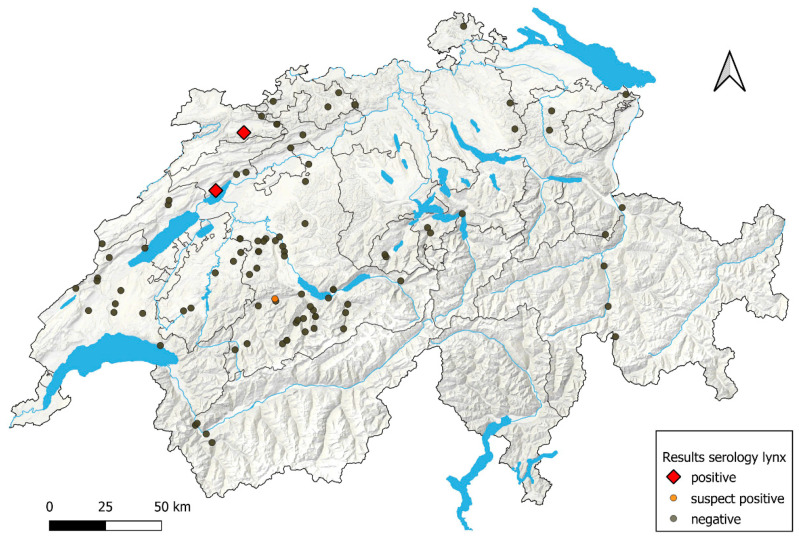
Distribution of serologically positive, suspect positive, and negative lynx in the different cantons of Switzerland and Liechtenstein. Grey shades show land features, blue indicates rivers and lakes, and grey lines mark canton boundaries on the QGIS-designed map. Each dot on the map represents one sampled animal.

The lynx F23_15 underwent sampling on two distinct occasions 1 week apart, revealing seroreactivity (suspect positive) in both S1 and Omicron S1-ELISA. Both samples were confirmed to have binding antibodies via iIFT. The lynx, captured in the Jura Mountains (Figure 6) for a relocation program, was identified as an adult female in good nutritional and health status.

Another lynx (W23_0441) positive for binding antibodies in the iIFT exhibited seroreactivity in sVNT (Figure 5), indicating the presence of neutralizing antibodies. Immunohistochemistry for the in situ detection of SARS-CoV-2 N, performed on formalin-fixed, paraffin-embedded lung samples, did not yield any evidence of in situ viral antigen expression. This particular lynx, less than a year old, had been discovered alone near human settlements (Figure 6) and was shot due to poor health, poor nutritional condition, and severe sarcoptic mange (*S. scabiei).*

#### 3.1.3. Wildcats

**Figure 7:**
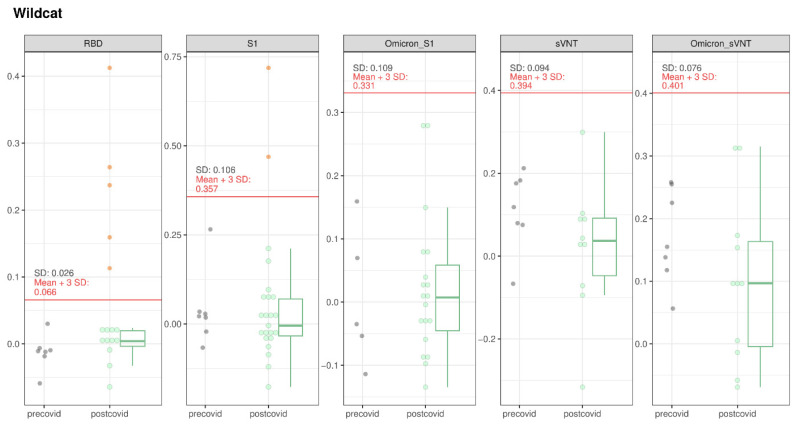
SARS-CoV-2 serological results for wildcats tested with RBD-, S1-, and Omicron S1-ELISA, as well as with sVNT and Omicron sVNT. The OD values of the different assays for the pre-COVID and post-COVID populations are represented. The boxes cover the 25th to 75th percentiles, while the whiskers represent the upper and lower 25th percentiles. For populations with less than 10 animals, values were plotted as points with a small jitter effect on the *y*-axis to prevent overplotting. When the population exceeded 10 animals, values were depicted using both dot plots and box plots. A cutoff, defined as the sum of the mean values for the pre-COVID population and three times the standard deviation, is denoted by a horizontal red line on the figure.

A total of 6 out of 24 wildcats tested suspect positive either in the RBD (n = 5), S1-ELISA (n = 2), or Omicron-S1-ELISA (n = 1). This resulted in a prevalence for suspect positive animals of 25% (95% CI 12.0–44.9%). Although some of the samples yielded relatively high ODs compared to the pre-COVID samples, none of them were confirmed by iIFT (Figure 7). One wildcat (T23_01) tested suspect positive in S1-ELISA and Omicron-S1-ELISA. However, due to limited serum availability, further confirmatory assays could not be conducted.

None of the samples tested positive in the sVNT or the Omicron sVNT. However, one wildcat (W22_9696), which tested suspect positive in the RBD-ELISA, was found to have neutralizing antibodies in PVNA. The highest neutralizing titers were observed for the SARS-CoV-2 variant BA.5. (Table S2). This male wildcat, aged over a year, was found deceased, presumably due to a collision with a car, in the canton of Vaud, approximately 200 m from human settlements.

With one positive case among the 24 samples, the observed prevalence is 4.2% (95% CI: 0.2–20.2%).

#### 3.1.4. Wolves and Golden Jackal

**Figure 8:**
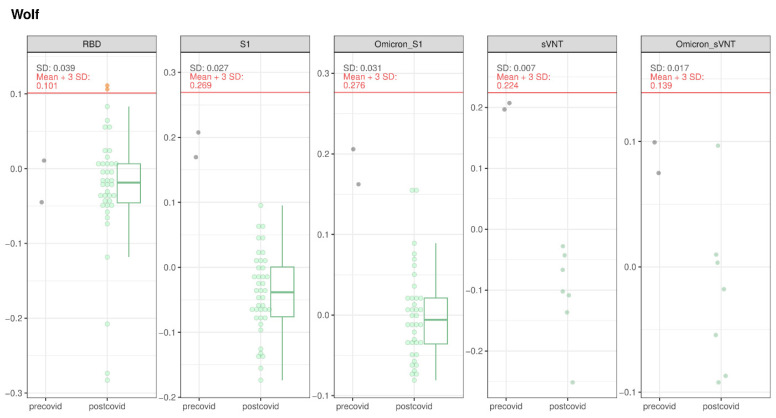
SARS-CoV-2 serological results for wolves tested with RBD-, S1-, and Omicron S1-ELISA, as well as with sVNT and Omicron sVNT. The OD values of the different assays for the pre-COVID and post-COVID populations are represented. The boxes cover the 25th to 75th percentiles, while the whiskers represent the upper and lower 25th percentiles. For populations with less than 10 animals, values were plotted as points with a small jitter effect on the *y*-axis to prevent overplotting. When the population exceeded 10 animals, values were depicted using both dot plots and box plots. A cutoff, defined as the sum of the mean values for the pre-COVID population and three times the standard deviation, is denoted by a horizontal red line on the figure.

Two wolves (2/41) were suspect positive in the RBD-ELISA (4.9%, 95% CI: 1.3–16.1%), but they both tested negative by iIFT. All sVNT and PVNA results for wolves were negative (Figure 8). The single golden jackal tested was found negative in RBD-ELISA and S1-ELISA.

#### 3.1.5. Mustelids

**Figure 9:**
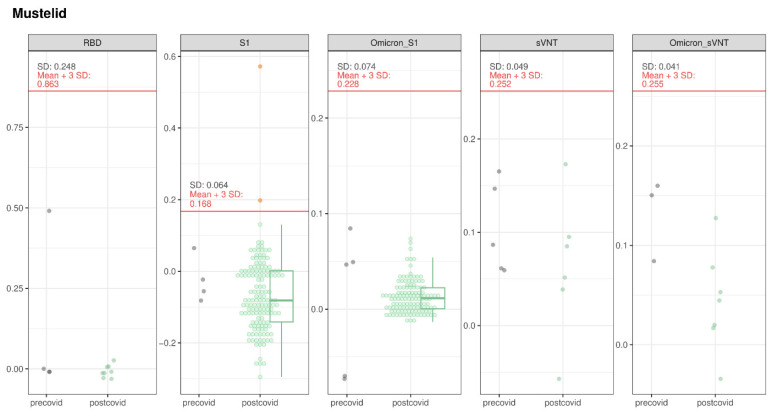
SARS-CoV-2 serological results for mustelids tested with RBD-, S1-, and Omicron S1-ELISA, as well as with sVNT and Omicron sVNT. The OD values of the different assays for the pre-COVID and post-COVID populations are represented. The boxes cover the 25th to 75th percentiles, while the whiskers represent the upper and lower 25th percentiles. For populations with less than 10 animals, values were plotted as points with a small jitter effect on the *y*-axis to prevent overplotting. When the population exceeded 10 animals, values were depicted using both dot plots and box plots. A cutoff, defined as the sum of the mean values for the pre-COVID population and three times the standard deviation, is denoted by a horizontal red line on the figure.

One polecat (1/10) and one stone marten (1/51) tested suspect positive in the S1-ELISA; both tested negative by RBD-ELISA, iIFT, sVNT, and PVNA. This resulted in a prevalence for suspect positive animals of 10% (95% CI: 5.1–40.4%) for polecats and of 2.0% (95% CI: 0.1–10.3%) for stone marten. One of the pre-COVID mustelids tested showed high OD values in the RBD-ELISA (Figure 9). There was insufficient sample material for further confirmatory testing.

### 3.2. Detection of Viral RNA

All 579 samples (210 oral swabs, 207 rectal swabs, 162 lung tissues) from the 175 necropsied animals and the 45 captured animals (for details see Table 2) tested negative in RT-qPCR.

## 4. Discussion

The study provides the first evidence of SARS-CoV-2 infections in wild, free-ranging red foxes, European wildcats, and Eurasian lynx.

Among the 446 tested foxes, 12 were confirmed to have binding antibodies, while 2 of them, along with 2 additional animals, exhibited neutralizing serological activity. The last two animals (#1577 sampled in November 2022 and #371 sampled in February 2023) showed the highest neutralization titers for Omicron BA.2 and Omicron BA.1, respectively, fitting the timeline of the emergence of the variants in humans in Switzerland [37]. Although all foxes lived near human settlements, they originated from distinct geographic areas across eight different cantons. It is worth noting that the vast majority of foxes, including those that tested positive, were sampled within 500 m of human settlements.

The geographical distribution of antibody-positive red foxes strongly indicated either multiple direct or indirect independent transmissions from humans, human waste, domesticated animals, or other wild animals to the fox population. This implies that the boundaries between humans and wild animals are permeable for a virus with a broad host range. Our results are in line with another study conducted in Virginia and Washington D.C. (USA) in 2022–2023. SARS-CoV-2 was detected in six wildlife species (deer mouse, Virginia opossum, raccoon, marmot, eastern cottontail, and eastern red bat), with seroprevalence three times higher in animals in areas with high human activity. Genomic analysis of the virus from wild animals was consistent with several Omicron variants circulating in humans, suggesting recent human-to-animal transmission [38]. This finding could also be relevant for other infectious diseases, such as Highly Pathogenic Avian Influenza (HPAI), which has been detected in wild red foxes in the Netherlands [39].

The data collected do not allow for any clear conclusions regarding the exact route of transmission. Factors such as proximity to human settlements, contact with human waste, food sources for wildlife, and the sharing of habitats with pets susceptible to infection, such as domestic cats and dogs, can shape the transmission routes. The risk of SARS-CoV-2 transmission through food sources, such as contaminated prey or other food waste, is rather low due to the high dose required for infection via oral transmission [40]. However, the ability of SARS-CoV-2 to survive in various conditions (low temperatures and protein, lipid-rich food) indicates that there remains a potential for transmission [40].

Remarkably, two lynx were also serologically positive, indicating that they had undergone SARS-CoV-2 infection. As the two positive animals in the present study had never been captured before, spillover from an infected wildlife professional during previous immobilizations can be ruled out. Lynx are solitary, live in rural areas, and feed mainly on wild ungulates. Foxes and, rarely, domestic animals might also be preyed upon by lynx [41,42]. Virus transmission is conceivable while killing and feeding on prey. Infection at marking sites where lynx typically rub their faces would also be an option. Monitoring with photo traps has shown that such sites are sometimes also visited by domestic cats. Inter-species transmissions of SARS-CoV-2 have been described between mink and domestic cats [43].

The adult female lynx F23_15, which tested positive in serology, was the only animal sampled twice, at two different time points. The initial sample was obtained during the animal’s capture for relocation measures, after which it was quarantined and sampled again just before release, one week later. Both samples exhibited similar levels of seropositivity in S1-ELISA and Omicron-S1-ELISA. The juvenile lynx, W23_0441, confirmed positive for binding antibodies and also showed neutralizing antibodies in sVNT testing. Discovered near settlements, the young lynx was euthanized due to poor health, malnutrition, and severe sarcoptic mange. The negative RT-qPCR and immunohistochemistry results do not support the hypothesis of a link between the infection and the poor state of health. However, sick young lynx that have lost their mother often search for easy food near settlements, which increases their risk of SARS-CoV-2 infection.

Given the high susceptibility of many felids, including domestic cats, to SARS-CoV-2, it was anticipated that the closely related Eurasian wildcat might also be susceptible. One wildcat (T23_01) tested suspect positive in S1-ELISA and Omicron-S1-ELISA, but no anamnestic details were available for this animal, and due to limited serum quantity, further testing of the suspect positive sample was not possible. One wildcat (W22_9696) tested positive in PVNA, showing the highest inhibition titer for the BA.5 variant. The animal was sampled in December 2022, aligning with the emergence of the BA.5 variant in humans. From June to November 2022, this variant was the most frequently detected in humans in Switzerland [37].

Concerning the wolves, two animals tested suspect positive for binding antibodies in RBD-ELISA, but the result could not be validated through further testing. So far, only one other wolf has been tested in Switzerland besides in the present study; the animal was a zoo-kept wolf, and it tested negative by RT-qPCR [14].

For mustelids, evidence of natural SARS-CoV-2 infection emerged in a study on wild Eurasian river otters (*Lutra lutra*) in Spain; the authors of the study detected SARS-CoV-2 RNA via RT-qPCR and confirmed the finding by sequencing [44]. In a separate study in Spain, two out of thirteen wild American mink (*Mustela vison*) tested positive for SARS-CoV-2 RNA, with the authors suggesting potential wastewater-related transmission in rural areas with high numbers of COVID-19 cases [45]. Notably, in our investigation, all mustelids (badger, stone marten, pine marten, and polecat) tested negative in confirmatory testing, with only one stone marten and one polecat showing suspect positivity in ELISA tests. Unlike otters, the mustelids tested in our study do not live in aquatic environments. They may therefore have a lower risk of exposure to viruses circulating in human populations.

The limited volume of some blood samples and the need to conduct multiple tests resulted in an inability to perform further validation tests on certain animals initially identified as seropositive via ELISA. Consequently, the prevalence of confirmed seropositive animals might be underestimated. Some animals were classified as suspect positive, despite the inability to confirm the result due to lack of material.

Nevertheless, we consider testing with multiple assays a necessity to assess proper seropositivity and avoid missing any positive samples. Considering the potential for missing positive cases, we conducted a broad ELISA panel to minimize the chance of overlooking seropositive animals.

The distinction between binding and neutralizing antibodies should be noted, as they do not necessarily align; this study detected animals with only one type of antibody or the other.

Due to the scarcity of pre-COVID reference samples for mustelids, wolves, and wildcats, validation of the serological tests was challenging. Comparisons with domestic species such as pre-COVID dogs and cats were initially considered for serological testing. However, we focused on the exclusive use of samples from wild species, such as wolves and wildcats, to ensure that the serological data accurately reflected the specific immune responses of these species, thereby omitting potential confounding factors potentially introduced by the samples from domestic animals.

Another study tested for potential cross-reactivity of samples from domestic cats with antibodies to the feline coronavirus (FCoV), an alpha coronavirus, to SARS-CoV-2, which is a betacoronavirus: 24 feline convenience serum samples with antibodies to FCoV as tested by immunofluorescence [46] were assessed for RBD-ELISA [30]. Twenty-three of these were negative, and one was seroreactive just over the set cut-off. An overall correlation between IFA titer and OD value in RBD-ELISA could not be found. The potential cross-reactivity with the little-described Fox Coronavirus (FoxCoV) [47] requires more research, which was hindered by the unavailability of blood samples for testing in our study.

Regarding the PVNA tests, the unexpected similarity in titers observed across several SARS-CoV-2 variants prompted further investigation into potential factors that influenced the neutralization patterns. The control test (using a VSV glycoproteins pseudotype) aimed to validate the usually similar titers observed in the neutralization assay and to check that factors like sample toxicity to the assay cell cultures were not responsible. The use of VSV glycoproteins, commonly found in larger animals like horses and cattle, served as a control to confirm the absence of false positive results in the canine samples. Only the samples that tested negative in the control assay were included in our analysis. Possible explanations for the similarity in titers include the existence of cross-neutralizing antibodies induced by another coronavirus, particularly in canids, or the presence of a broader, non-variant-specific SARS-CoV-2 antibody response in certain animals. Additionally, potential alterations in serum quality due to complications in long custom clearance procedures are to be considered.

Despite the diversity regarding the geography, human population density, and wildlife management in Switzerland, this study enabled an estimation of the national seroprevalence in wild animals at risk of exposure and infection.

While we achieved our sample size goals for wild felids and wolves, and nearly reached our target for foxes, we aimed to collect 430 samples from mustelids. Unfortunately, we could only gather 147 samples due to low submission rates for these small to medium-sized mammals. Many samples originated from roadkill or culled animals, and only a limited amount of blood was available in these cases, which complicates sampling and might account for the low submission rate compared to that for larger species such as the red fox.

SARS-CoV-2 RNA was not detected in the sample analyzed using a sensitive RT-qPCR with two different targets. Thus, no active infection could be proven in any cases at the time of sampling. Therefore, no statements about the clinical significance, clinical signs, virus excretion, or prognosis of SARS-CoV-2 infections in wild animals were possible. However, the detection of antibodies and the absence of the viral genome allow us to conclude that the tested wild foxes, wildcat, and lynx had survived the infection and eliminated the virus.

## 5. Conclusions

Serological surveillance is a valuable tool in assessing SARS-CoV-2 susceptibility across animal species. While evidence of viral infection was found in 2% to 4% of Swiss red foxes, European wildcats, and Eurasian lynx through antibody detection, no active infection (viral RNA) was detected. This study provides the first confirmed seropositive testing of these species in free-ranging populations worldwide. The absence of discernible geographical patterns suggested multiple sporadic spillover events of unknown origin. To reduce the possible potential transmission of anthropozoonotic diseases and ensure the safety of animals and humans, the implementation of strict protective measures when handling wildlife is essential.

## Figures and Tables

**Table 1:** Species and number of all animals sampled within this study, using different sampling opportunities, with calculated/estimated sample sizes for each species, their characteristic behavioral patterns, and protection status.

Taxonomy	Species	Behavior	Protected	Necropsies ^1^	Field Sampling	Immobilization	Total/Estimated Sample Sizes
Canids	Red fox	Social	No	30	419		449/600
	Gray wolf	Social	Yes	41			41/20
	Golden jackal	Variable	Yes	1			1/<5
Felids	Eurasian lynx	Solitary	Yes	56		39	95/60
	European wildcat	Solitary	Yes	18		6	24/10
Mustelids	Stone and pine marten	Solitary	No	20	44		64/210
	European badger	Social	No	8	65		73/210
	European polecat	Solitary	Yes	2	8		10/10
Total	8			176	536	45	757/1115

^1^ Animals tested by serology and RT-qPCR.

**Table 2:** Number and type of samples collected within this study between 2020 and 2023.

Taxonomy	Species	Serum	Oronasal/ Oropharyngeal Swabs	Rectal Swabs	Lung Tissue
Canids	Red fox	446	29	27	29
	Gray wolf	41	39	38	36
	Golden jackal	1	1	1	1
Felids	Eurasian lynx	92 *	91	91	52
	European wildcat	23	22	22	16
Mustelids	European marten	62	18	18	18
	European badger	72	8	8	8
	European polecat	10	2	2	2
Total	8	747	210	207	162

* one animal was sampled and tested twice.

**Table 3:** Overview of binding antibodies and neutralization results in all tested samples.

Species	No of Samples	Suspect Positive Binding Antibodies	IFT Confirmed Binding Antibodies °	sVNT Positive °	PVNA Positive °
Red fox	446	64/446	12/64	0/116	2/120
Grey wolf	41	2/41	0/7	0/7	0/7
Golden jackal	1	0/1	nt*	nt*	nt*
Eurasian lynx	92 *	4 */92	3 */11	1/16 *	0 */10
European wildcat	24	6/24	0 °/7	0 °/11	1 °/11
Stone marten	48	1/48	0/1	0/4	0/1
Pine marten	13	0/13	nt*	nt*	nt*
European badger	72	0/72	nt*	nt*	nt*
European polecat	10	1/10	0/4	0/2	0/2

* a total of 1 animal was tested twice; ° for some confirmation assays not enough material was available; nt*: not tested.

**Table 4:** Seropositive red foxes confirmed for binding antibodies through iIFT and/or neutralizing antibodies through PVNA or sVNT.

Fox ID	Sampling Date	Sex	Age	Clinical Signs	Localization	Binding/Neutralizing
80	3 December 2021	Female	Over a year	Sarcoptic mange	Canton Nidwalden, near dogs	Binding
811	27 January 2022	Male	Over a year	Wounded right foreleg	Canton Bern, near cattle	Binding
943	31 January 2022	Unknown	Over a year	Apparently healthy	Canton Valais	Binding
W22_6633	26 April 2022	Male	Over a year	Signs of canine distemper (CDV) *	Canton Zurich	Binding
1091	11 November 2022	Female	Over a year	Apparently healthy	Canton Zurich	Binding
1434	12 November 2022	Male	Over a year	Unknown	Canton Zurich	Binding
1787	8 December 2022	Unknown	Over a year	Apparently healthy	Canton St. Gallen	Binding
866	5 January 2023	Female	Under a year	Apparently healthy	Canton Ticino	Binding
1655	16 January 2023	Male	Over a year	Hit by car, apparently healthy	Canton Fribourg	Binding
1827	23 February 2023	Female	Over a year	Apparently healthy	Canton Ticino	Binding
1501	14 March 2023	Male	Over a year	Apparently healthy	Canton Vaud	Binding
1761	29 March 2023	Male	Over a year	Apparently healthy	Canton Valais	Binding
1577	9 November 2022	Male	Over a year	Apparently healthy	Canton Zurich, near sheep	Neutralizing
371	28 February 2023	Male	Over a year	Suspected signs of distemper (CDV)	Canton St. Gallen	Neutralizing

* Clinical signs and pathological lesions consistent with Canine Distemper Virus (CDV) infection, brain tissue positive for viral RNA (CDV PCR).

## Data Availability

All available data are presented in this manuscript.

## Supplementary Materials

The following supporting information can be downloaded at: https://www.mdpi.com/article/10.3390/v16091407/s1, Figure S1: Indirect Immunofluorescence test (iIFT). (a) Seropositive sample from fox 943 on infected cells; (b) Seropositive sample from fox 943 on non-infected cells.; Table S1: Overview of antibody binding and virus neutralization results in suspect positive and positive animals. Samples with ODs above the cutoff values (≥mean+3SD) and those exhibiting high ODs under the cutoff values (≥mean+2SD) are marked in bold. The cut-off for IFA was 1:8; the highest titers in iIFT are marked in bold. Table S2: PVNA positive animals and corresponding variants. The highest neutralizing titer is marked in bold. Table S3: Distribution of sampled animals across Cantons of Switzerland and in the Principality of Liechtenstein.
