# Supplementary material for: Investigations on the Potential Role of Free-Ranging Wildlife as a Reservoir of SARS-CoV-2 in Switzerland
Source: Viruses. 2024 Sep 3;16(9):1407. doi: 10.3390/v16091407 (PMC11437421; doi:10.3390/v16091407)
Supplement: Supplementary file 1 [file viruses-16-01407-s001.zip › viruses-3170294-supplementary.pdf]

Supplementary

Table S1: Overview of antibody binding and virus neutralization results in suspect positive and positive animals. Samples with ODs above the cutoff values (≥ mean+3SD) and those exhibiting high ODs under the cutoff values (≥ mean+2SD) are marked in bold. The cut-off for IFA was 1:8; the highest titers in iIFT are marked in bold.

**Sample ID**	**Sampling date**	**Species**	**RBD-ELISA(%)**	**S1-ELISA (%)**	**Omicron S1-ELISA (%)**	**iIFT (titer)**	**sVNT (%)**	**Omicron sVNT (%)**	**PVNA**	**Interpretation**
**777**	10.11.2021	Fox	**66.1**	-4.8		< 1/8	-6.7		Negative	Suspect positive
**80**	03.12.2021	Fox	**31.5**	-1.2	-5.1	**1/16**	-4.6		Negative	**Binding activity**
**468**	13.12.2021	Fox	-4.1	**18.5**	**13.8**	< 1/8	7.2		Negative	Suspect positive
**152**	18.12.2021	Fox	18.4	**7.6**	5.9	< 1/8	-5.0		Negative	Suspect positive
**446**	19.12.2021	Fox	**49.3**	0.2	6.7	< 1/8	-4.4		Negative	Suspect positive
**445**	19.12.2021	Fox	-9.9	**12.7**	2.3	< 1/8	7.3		Negative	Suspect positive
**67**	23.12.2021	Fox	-14.8	**12.4**	**18.5**	< 1/8	7.7		Negative	Suspect positive
**497**	23.12.2021	Fox	-29.4	**16.7**		< 1/8	-10.7		nt*	Suspect positive
**533**	02.01.2022	Fox	17.9	-3.3	**16.7**	<1/8	-1.1	1.0	Negative	Suspect positive
**703**	06.01.2022	Fox	-1.2	-0.4	**12.4**	<1/8	14.1	10.0	Negative	Suspect positive
**494**	18.01.2022	Fox	-22.4	**13.9**	5.3	< 1/8	2.0	4.4	Negative	Suspect positive
**454**	19.01.2022	Fox	-8.3	**20.2**	**10.8**	< 1/8	3.1	-15.8	Negative	Suspect positive
**855**	25.01.2022	Fox	-20.9	**18.2**	5.2	< 1/8	10.7	-6.7	Negative	Suspect positive
**805**	26.01.2022	Fox	-5.9	1.0	**12.2**	<1/8	14.5	14.0	Negative	Suspect positive
**770**	26.01.2022	Fox	-6.1	-3.1	**12.2**	<1/8	-7.5	1.3	Negative	Suspect positive
**815**	27.01.2022	Fox	**29.4**	-0.2	**17.6**	<1/8	6.0	15.7	Negative	Suspect positive
**806**	27.01.2022	Fox	-7.7	-3.5	**12.4**	<1/8	12.6	11.0	Negative	Suspect positive
**811**	27.01.2022	Fox	13.9	-2.2	**12.0**	**1/16**	-13.7	2.2	Negative	**Binding activity**
**828**	29.01.2022	Fox	-18.5	**9.1**	**19.3**	< 1/8	7.0	0.4	Negative	Suspect positive
**943**	31.01.2022	Fox	-19.4	**7.8**	**7.9**	**1/64**	6.3	4.8	Negative	**Binding activity**

**458**	02.02.2022	Fox	6.6	-2.5	**13.8**	nt*	8.6	22.8	nt*	Suspect positive
**467**	02.02.2022	Fox	-1.8	0.0	**16.7**	<1/8	0.0	22.9	Negative	Suspect positive
**W22_6633**	26.04.2022	Fox	**37.0**	0.6	6.7	**1/16**	8.8	1.1	Negative	**Binding activity**
**991**	07.07.2022	Fox	2.4	-3.7	**15.4**	<1/8	7.5	19.2	Negative	Suspect positive
**808**	16.07.2022	Fox	-0.3	-11.7	**17.0**	<1/8	-0.8	19.0	Negative	Suspect positive
**386**	23.07.2022	Fox	-2.8	-9.5	**15.7**	<1/8	5.8	19.8	Negative	Suspect positive
**1547**	15.10.2022	Fox	-8.6	-2.5	**14.2**	<1/8	-1.7	4.5	Negative	Suspect positive
**1134**	16.10.2022	Fox	-9.0	2.5	**14.9**	nt*	-0.1	19.9	nt*	Suspect positive
**1395**	27.10.2022	Fox	-22.3	**17.0**	**13.5**	< 1/8	3.6	11.7	Negative	Suspect positive
**1166**	01.11.2022	Fox	-15.4	-0.9	**13.5**	< 1/8	9.4	9.8	Negative	Suspect positive
**1373**	04.11.2022	Fox	0.3	5.6	**15.7**	<1/8	11.3	31.4	Negative	Suspect positive
**1241**	04.11.2022	Fox	-23.4	3.6	**20.9**	< 1/8	10.3	-7.8	Negative	Suspect positive
**1128**	04.11.2022	Fox	-21.4	5.3	**37.3**	nt*	-0.6	-13.1	nt*	Suspect positive
**1168**	04.11.2022	Fox	-14.5	-0.4	**12.8**	<1/8	2.6	9.2	Negative	Suspect positive
**1577**	09.11.2022	Fox	-3.8	-0.5	**10.0**	<1/8	6.3	10.0	**Positive**	**Neutralizing activity**
**1070**	09.11.2022	Fox	-2.6	3.5	**18.0**	< 1/8	13.2	12.6	Negative	Suspect positive
**1091**	11.11.2022	Fox	-24.6	**94.5**	2.4	**1/32**	4.4	-0.3	Negative	**Binding activity**
**1434**	12.11.2022	Fox	-24.6	2.1	**21.1**	**1/512**	9.5	3.4	Negative	**Binding activity**
**1136**	16.11.2022	Fox	-25.6	4.5	**14.4**	< 1/8	14.2	2.8	Negative	**Binding activity**
**1006**	21.11.2022	Fox	**37.8**	-10.4	-5.2	< 1/8	-18.4	-1.9	Negative	Suspect positive
**1287**	26.11.2022	Fox	7.8	-5.4	**12.8**	<1/8	9.7	20.5	Negative	Suspect positive
**1787**	08.12.2022	Fox	**39.7**	-6.6	**34.9**	**1/16**	13.7	**33.9**	Negative	**Binding and neutralizing activity**
**1167**	12.12.2022	Fox	-6.3	-0.9	**33.1**	< 1/8	8.8	28.9	Negative	Suspect positive
**1664**	12.12.2022	Fox	-10.8	-6.1	**12.5**	<1/8	-9.7	9.2	Negative	Suspect positive
**923**	26.12.2022	Fox	-1.0	-5.4	**16.5**	<1/16	7.3	24.6	Negative	Suspect positive

**1724**	27.12.2022	Fox	-5.8	5.4	**20.7**	< 1/8	12.4	21.8	Negative	Suspect positive
**1439**	28.12.2022	Fox	-28.1	0.7	**38.3**	< 1/8	9.0	-8.4	Negative	Suspect positive
**333**	28.12.2022	Fox	-28.3	2.4	**21.2**	< 1/8	5.1	14.3	Negative	Suspect positive
**1120**	28.12.2022	Fox	-11.5	1.8	**20.3**	< 1/8	-3.3	10.5	Negative	Suspect positive
**1106**	28.12.2022	Fox	-7.1	-3.8	**12.8**	<1/8	-3.4	17.8	Negative	Suspect positive
**1746**	29.12.2022	Fox	-8.9	-6.1	**15.9**	<1/16	10.9	16.2	Negative	Suspect positive
**1225**	29.12.2022	Fox	-11.4	-4.8	**12.6**	<1/8	12.8	18.1	Negative	Suspect positive
**1325**	02.01.2023	Fox	-27.4	1.5	**38.7**	< 1/8	10.3	-2.8	Negative	Suspect positive
**1393**	03.01.2023	Fox	-7.7	-4.5	**17.4**	<1/8	10.7	24.3	Negative	Suspect positive
**1460**	04.01.2023	Fox	-18.8	1.9	**35.1**	< 1/8	9.3	-5.7	Negative	Suspect positive
**1458**	04.01.2023	Fox	-2.9	-2.0	**25.9**	< 1/8	11.7	28.0	Negative	Suspect positive
**863**	04.01.2023	Fox	-3.3	-4.1	**18.6**	<1/8	7.4	19.2	Negative	Suspect positive
**879**	05.01.2023	Fox	-10.8	-4.5	**12.3**	<1/8	-0.4	9.1	Negative	Suspect positive
**866**	05.01.2023	Fox	-12.8	-4.2	**16.7**	**1/16**	12.0	23.7	Negative	**Binding activity**
**1652**	11.01.2023	Fox	-11.8	-5.0	**28.1**	< 1/8	5.9	19.6	Negative	Suspect positive
**1654**	11.01.2023	Fox	3.7	-6.0	**12.0**	<1/8	0.8	-12.2	Negative	Suspect positive
**854**	11.01.2023	Fox	-5.3	-5.1	**12.7**	<1/8	-9.4	14.7	Negative	Suspect positive
**1655**	16.01.2023	Fox	14.8	**74.1**	**33.0**	**1/32**	17.7	19.6	Negative	**Binding activity**
**345**	25.01.2023	Fox	**52.6**	-2.4	1.6	< 1/8	8.0	0.4	Negative	Suspect positive
**1852**	02.02.2023	Fox	-3.0	**59.4**	-4.6	< 1/8	12.0	24.5	Negative	Suspect positive
**1858**	02.02.2023	Fox	-4.2	5.1	**13.3**	<1/8	7.2	31.5	Negative	Suspect positive
**1583**	03.02.2023	Fox	-7.0	**14.5**	**8.3**	< 1/8	-10.0	5.0	Negative	Suspect positive
**1827**	23.02.2023	Fox	-19.1	-0.1	**10.8**	**1/16**	-4.2	16.2	Negative	**Binding activity**
**371**	28.02.2023	Fox	-20.1	0.8	**8.4**	<1/8	8.3	17.2	**Positive**	**Neutralizing activity**
**1501**	14.03.2023	Fox	-18.0	3.4	**7.8**	**1/16**	-17.3	7.8	Negative	**Binding activity**

**1761**	29.03.2023	Fox	**97.9**	**120.4**	**16.9**	1/8	10.0	21.9	Negative	Suspect positive
**F22_21_Dawn**	11.04.2022	Lynx	8.7	**58.8**	**20.9**	<1/8	2.6	-4.2	Negative	Suspect positive
**W23_0441**	22.02.2023	Lynx	0.5	**80.1**	**48.0**	**1/8**	**29.0**	**34.2**	Negative	**Binding and neutralizing activity**
**F23_15_Jura**	23.02.2023	Lynx	-6.8	**39.7**	**33.5**	**1/32**	-10.6	-3.4	Negative	**Binding activity**
**W21_4826**	10.12.2021	Wildcat	**41.3**	-12.0	-9.7	<1/8	-9.5	17.3	Negative	Suspect positive
**W22_6267**	29.03.2022	Wildcat	**23.7**	1.8	-8.7	nt*	29.9	9.9	nt*	Suspect positive
**W22_9325**	02.12.2022	Wildcat	**11.3**	-6.4	-5.9	< 1/8	4.3	9.4	Negative	Suspect positive
**W22_9696**	15.12.2022	Wildcat	**15.9**	7.0	15.0	< 1/8	8.4	-1.4	**Positive**	**Neutralizing activity**
**F23_2_Lenny**	16.01.2023	Wildcat	**26.4**	**46.9**	**27.8**	<1/8	-31.6	0.5	Negative	Suspect positive
**T23_01_Wildkatze_Kurs**	Not kwown	Wildcat		**71.9**	**28.0**	nt*	nt*	nt*	nt*	Suspect positive
**W22_6139**	21.03.2022	Wolf	**11.1**	-6.0	2.1	<1/8	-6.7	-9.2	Negative	Suspect positive
**W22_9272**	09.11.2022	Wolf	**10.6**	-8.0	0.0	< 1/8	-25.2	1.0	Negative	Suspect positive
**23**	20.11.2021	Polecat	0.8	**19.8**		< 1/8	5.1	1.7	Negative	Suspect positive
**12**	25.05.2022	Stone marten	0.6	**57.2**	3.0	< 1/8	8.5	7.8	Negative	Suspect positive

nt*: not tested, no more material available

Table S2: PVNA positive animals and corresponding variants. The highest neutralizing titer is marked in bold.

Sample ID	Wuhan titer	Alpha titer	Delta titer	Omicron titer	BA.2 titer	BA.5 titer	BQ.1.1 titer	XBB titer
**Fox 1577**	67	68	65	68	**78**	63	53	72
**Fox 371**	≤50	≤50	≤50	**71**	66	≤50	≤50	≤50
**Wildcat W22_9696**	58	58	56	≤50	107	**135**	98	117

Table S3: Distribution of sampled animals across Cantons of Switzerland and in the Principality of Liechtenstein

Localization	Number of all sampled animals (all species)
Fribourg	118
Solothurn	11
Aargau	8
Appenzell Ausserrhoden	8
Bern	129
Basel-Landschaft	9
Geneva	8
Glarus	2
Grisons	34

Jura	4
Lucerne	8
Neuchâtel	50
Nidwalden	3
Obwalden	16
St. Gallen	15
Schaffhausen	6
Schwyz	8
Thurgau	4
Ticino	22
Uri	8
Vaud	72
Valais	51
Zug	1
Zurich	151
Principality of Liechtenstein	5
Not known	5

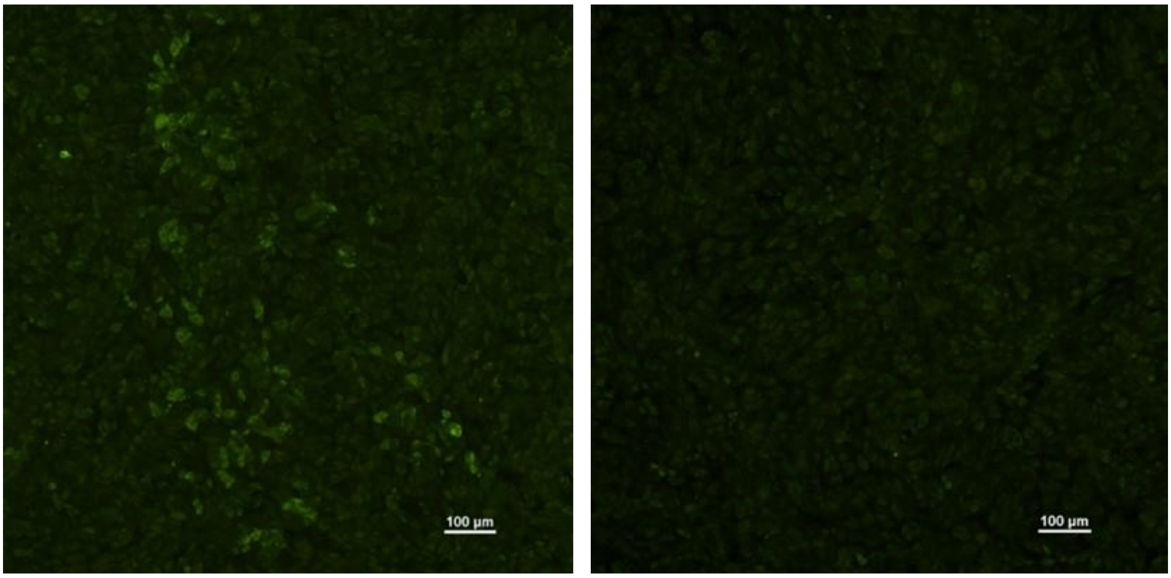

(**a**) (**b**)

Figure S1: Indirect Immunofluorescence test (iIFT). (**a**) Seropositive sample from fox 943 on infected cells; (**b**) Seropositive sample from fox 943 on non-infected cells.
